# Synapses, Microglia, and Lipids in Alzheimer’s Disease

**DOI:** 10.3389/fnins.2021.778822

**Published:** 2022-01-12

**Authors:** Patrick J. Paasila, Jason A. Aramideh, Greg T. Sutherland, Manuel B. Graeber

**Affiliations:** ^1^Charles Perkins Centre, School of Medical Sciences, Faculty of Medicine and Health, The University of Sydney, Camperdown, NSW, Australia; ^2^School of Medicine, Western Sydney University, Campbelltown, NSW, Australia; ^3^Brain and Mind Centre, Faculty of Medicine and Health, The University of Sydney, Camperdown, NSW, Australia

**Keywords:** Alzheimer’s disease, APOE, lipids, microglia, synapses, TREM2

## Abstract

Alzheimer’s disease (AD) is characterised by synaptic dysfunction accompanied by the microscopically visible accumulation of pathological protein deposits and cellular dystrophy involving both neurons and glia. Late-stage AD shows pronounced loss of synapses and neurons across several differentially affected brain regions. Recent studies of advanced AD using post-mortem brain samples have demonstrated the direct involvement of microglia in synaptic changes. Variants of the Apolipoprotein E and Triggering Receptors Expressed on Myeloid Cells gene represent important determinants of microglial activity but also of lipid metabolism in cells of the central nervous system. Here we review evidence that may help to explain how abnormal lipid metabolism, microglial activation, and synaptic pathophysiology are inter-related in AD.

## Introduction

Alzheimer’s disease (AD) accounts for 60–80% of total dementia diagnoses (Brookmeyer et al., 2011; Alzheimer’s Association, 2021). Extracellular β amyloid (Aβ) plaques and intraneuronal neurofibrillary tangles (NFTs) are the major pathological characteristics of AD. The gold-standard for a definitive diagnosis of AD is post-mortem neuropathology (Hyman et al., 2012; Montine et al., 2012). However fluid analytes—including most recently the development of the serum phosphorylated tau (threonine 217) blood test (Barthélemy et al., 2020)—and imaging biomarkers allow for a reasonably confident diagnosis to be made *in vivo* (Jack et al., 2010; Leuzy et al., 2018).

Synapses are the sites at which neuronal communication takes place by chemical or electrical means (Alcamí and Pereda, 2019). Synaptic plasticity refers to the brain’s ability to modify neural circuitry structurally and functionally at the synaptic level to facilitate learning, memory, cognition, and the regulation of emotions and behaviours (Kennedy, 2016)—though there remains significant debate in the field as to the sole importance of synapses in these processes, particularly memory (Trettenbrein, 2016). Long-term potentiation (LTP) and long-term depression (LTD) represent well researched mechanisms of synaptic plasticity which entail long lasting and activity dependent changes in synaptic efficacy as shown by *in vitro* and *in vivo* electrophysiological recordings (Abraham et al., 2019). Normal ageing is associated with a loss of synapses (Masliah et al., 1993a). This is markedly accelerated in AD which has been described as a disease of synaptic failure (Terry, 2000; Selkoe, 2002); albeit with the caveat that synaptic changes are not specific to AD (Scheff et al., 2014).

Microglia are the central nervous system’s (CNS’s) resident phagocytes of mesodermal origin (predominantly from blood islands of the embryological yolk sac) and as such share many characteristics with macrophages of myeloid lineage (Ginhoux and Prinz, 2015). Microglia perform a number of key functions in the CNS: (1) synaptogenesis (Miyamoto et al., 2016), synaptic pruning during development (Paolicelli et al., 2011), and complement-mediated elimination (Streit et al., 1988; Stevens et al., 2007; Schafer et al., 2012) in the adult brain that may represent a potential biological mechanism of forgetting (Wang et al., 2021); (2) vasculogenesis (Ronaldson and Davis, 2020); (3) constant surveillance of their local microenvironment (Nimmerjahn et al., 2005) by means of their sensome (Hickman et al., 2013), including continual monitoring of neuronal activity (Wake et al., 2013)—notably by exerting protective effects through somatic purinergic junctions (Cserép et al., 2020); (4) regulation of myelin homeostasis (Healy et al., 2016); (5) modification of synaptic plasticity through cytokine signalling and brain-derived neurotrophic factor (BDNF) (Wu et al., 2015); (6) innate immune functions (Kofler and Wiley, 2011), including convergence on sites of injury (Davalos et al., 2005), detection of danger-associated molecular patterns (alarmins and pathogen-associated molecular patterns) (Bianchi, 2007), phagocytosis (Sierra et al., 2013), macropinocytosis (Li et al., 2013), antigen presentation (Schetters et al., 2017), secretion of anti- and pro-inflammatory cytokines (Da Pozzo et al., 2019), and regulation of neuronal apoptosis in the setting of traumatic injury (Wang et al., 2020); and (7) coordination with astrocytes in aspects of each the aforementioned functions (Matejuk and Ransohoff, 2020).

Glia have long been implicated in AD since Alzheimer himself commented on their involvement in his original case report (Alzheimer, 1907)—“*The glia have formed plentiful fibres; in addition, many glial cells exhibit large fat sacks.*” A more detailed follow-up of microglia in AD began toward the end of the last century (Eikelenboom and Stam, 1982; Dickson et al., 1988; Eikelenboom et al., 1989; Itagaki et al., 1989; McGeer et al., 1989; Perlmutter et al., 1990; Sasaki et al., 1997). Since then, microglial involvement has been demonstrated in many neurodegenerative (Hickman et al., 2018) and psychiatric diseases (Tay et al., 2017), including AD, frontotemporal dementia, Parkinson’s disease, Huntington’s disease, motor neurone disease, prion diseases, chronic traumatic encephalopathy, bipolar disorder, major depressive disorder, and schizophrenia. More recently in AD, microglia have been shown to internalise greater quantities of synaptic material during the symptomatic phase of the disease. This has been demonstrated using confocal (Tzioras et al., 2019) and single-molecule localisation microscopy (Paasila et al., 2021) of archival cortical tissue samples.

Lipids are enriched in the CNS and figure prominently in AD pathophysiology (Di Paolo and Kim, 2011). Bozek et al. (2015) found that 75% of molecules from a panel of some 5713 lipids were either enriched or depleted in human brain compared to other mammals. Lipids participate in cell structure, cellular signalling, energy balance, and inflammatory signalling (Cermenati et al., 2015). For instance, synaptogenesis is promoted by glia-derived cholesterol (Mauch et al., 2001)—which also serves as the critical precursor compound of neuroactive steroids capable of altering gene expression and ultimately neuron survival (Melcangi et al., 2008). The importance of lipids to brain health is highlighted by genetic connections between neurological disease and cholesterol metabolism (Björkhem et al., 2010), and particularly in AD (Kunkle et al., 2019). Most classes of lipids are involved in the pathogenesis of AD, including cholesterols, sterols, glycerolipids, glycerophospholipids, and sphingolipids (Chew et al., 2020). The *APOE* ε4 allele represents the most direct genetic link between lipid metabolism and AD (Corder et al., 1993). *APOE* is highly expressed in the liver and brain where astrocytes are thought to be a main source (Grehan et al., 2001). APOE is involved in the transportation and metabolism of lipids, functioning as a ligand for low-density and very low-density lipoprotein (LDL and VLDL, respectively) receptors that trigger receptor-mediated endocytosis of lipoprotein particles (Bu, 2009). APOE appears to exert its effect on AD risk by influencing the time of disease onset as well as the total load of Aβ pathology (Kim et al., 2009a).

## Proteinopathy in Alzheimer’s Disease

### β Amyloid

β Amyloid (Aβ) is derived from the large type I transmembrane protein APP (Kang et al., 1987; Dyrks et al., 1988) found at both pre- and post-synapses and which is involved in dendritic spine plasticity (Montagna et al., 2017). APP undergoes a constant cycle of trafficking through the endomembrane system: it is first routed from the endoplasmic reticulum to the plasma membrane, Golgi apparatus, or *trans*-Golgi network (Haass et al., 2012). Nascent APP is post-translationally modified by glycosylation, phosphorylation, and sulphation. The small proportion of APP that reaches the plasma membrane is endocytosed within minutes and recycled or degraded in lysosomes. Our recently published results also demonstrate a mechanism of “short-circuited” APP recycling governed by lactoferrin which results in increased Aβ production (Tsatsanis et al., 2021). APP may be proteolytically cleaved by γ-secretase and either α- or β-secretase. β-secretase initiates the amyloidogenic pathway responsible for the generation of Aβ peptides. β-secretase cleaves APP in the extracellular domain to form the secreted APP ectodomain (APPsβ) and the membrane-bound APP carboxyl-terminal fragment (βCTF). βCTF is subsequently cleaved by γ-secretase within the plasma membrane (termed regulated intramembrane proteolysis; Lichtenthaler et al., 2011) or the endosomal-lysosomal system. Cleavage by γ-secretase can occur at several sites; labelled the ε-, ζ-, and γ-site, respectively. The γ-cleavage site varies in its position and is therefore responsible for the production of Aβ peptides of different lengths, from Aβ_37_ to Aβ_43_ (or longer). This is of relevance to AD as Aβ_39/40_ predominate in cerebral amyloid angiopathy (CAA) (Prelli et al., 1988; Suzuki et al., 1994) and Aβ_42/43_ in parenchymal deposits (Iwatsubo et al., 1994). The latter species are thought by some to constitute the neurotoxic oligomers responsible for initiating AD (Haass and Selkoe, 2007; Saito et al., 2011; Masters and Selkoe, 2012; Selkoe and Hardy, 2016).

Aggregates of Aβ filaments are observed as plaques in post-mortem AD brains (Walker, 2020). Morphological subtypes of Aβ plaques include diffuse (also, “primitive” or “immature”), fibrillar, dense-cored (also “classical” or “mature”), and burned-out (also, “core-only”) plaques. Other Aβ plaque types include subpial bands (Thal et al., 2000), cotton-wool plaques which feature prominently in *PSEN1* (also *PS1*; encoding presenilin 1) familial AD (Tabira et al., 2002), lake-like patches in the presubiculum (Wisniewski et al., 1998), and the recently described coarse-grained plaques in early onset AD (Boon et al., 2020). Neuritic plaques are a minority subset of Aβ plaques that are most often associated with phosphotau-positive dystrophic neurites. Aβ plaques occur in most elderly but are not universal (Braak et al., 2011; Jicha et al., 2012). Further, Aβ plaques are not sufficient to cause AD, however there is a strong association between their formation and the eventual development of AD as demonstrated by rare mutations (including *APP*, *PSEN1*, and *PSEN2*) in familial early- and late-onset AD (Cruchaga et al., 2012; Sassi et al., 2014; Lanoiselée et al., 2017).

### Microtubule Associated Protein Tau

A hyperphosphorylated form of the microtubule associated protein tau (MAPT) (“tau” for short) is the subunit of NFTs, neuropil threads, and tau positive dystrophic neurites (Mandelkow and Mandelkow, 2012)—collectively referred to as neurofibrillary degeneration (NFD) (Iqbal et al., 2010b). Tau is a member of the type 2 microtubule associated protein (MAP) family. It is a highly soluble protein which is unfolded in its native state (Schweers et al., 1994; Mukrasch et al., 2009). It is expressed as six alternatively spliced isoforms with 0, 1, or 2 amino (N-) terminal inserts of 29 residues each (0N, 1N, or 2N; derived from exons 2 and 3) and 3 or 4 carboxyl (C-) terminal repeats of 31–32 residues (3R or 4R; derived from exon 10) (Goedert et al., 1989). The full-length protein can be divided into an N-terminal “projection domain,” which is directed away from the microtubule, followed by a C-terminal “assembly domain.” The assembly domain is further subdivided into a proline-rich region, the microtubule-binding repeats, and a C-terminal tail (Mandelkow and Mandelkow, 2012). Tau contains 85 potential serine, threonine, and tyrosine phosphorylation sites, most of which reside in the proline-rich region and the C-terminal tail (Noble et al., 2013). Tau exhibits many structural conformations, biochemical modifications, and the ability to interact with several different protein types, including chaperones, cytoskeletal proteins, kinases, motors, and phosphatases (Mandelkow and Mandelkow, 2012). However, unlike *APP*, mutations in *MAPT* cause frontotemporal dementia but not AD.

Tau is susceptible to a range of disease-related processes, including acetylation, glycation, glycosylation, methylation, nitration, oxidation, ubiquitination, truncation, and missorting to the somatodendritic compartment (Ittner and Ittner, 2018). Aggregations of phosphotau occur first as granular oligomers which then aggregate further to form fibrils (Maeda et al., 2007). Soluble oligomers form early in the pathogenesis of AD and may represent the major neurotoxic substrate (Lasagna-Reeves et al., 2012; Ward et al., 2012) and which are thought by some to initiate the AD pathophysiological process rather than Aβ (Arnsten et al., 2021). Conversely, intraneuronal, insoluble fibrillar tau inclusions may represent end-stage lesions, appearing to be inert markers of earlier pathological changes to soluble tau species (Iqbal et al., 2010a). Importantly, the density and anatomical spread of NFTs remain criteria for diagnostic and staging purposes (Montine et al., 2012).

## Synapses in Alzheimer’s Disease

The overall picture is that of a widespread loss of synapses and synaptic proteins in AD (Terry and Davies, 1980; Davies et al., 1987; Masliah et al., 1989). During the symptomatic end-stage of AD the pronounced loss of synapses correlates with the severity of symptoms (DeKosky and Scheff, 1990; Terry et al., 1991; Masliah et al., 1993b; Dickson et al., 1995; Sze et al., 1997). A series of studies demonstrated this using electron microscopy (EM) (Scheff and Price, 2003) on the hippocampal dentate gyrus (Scheff et al., 1996, 2006; Scheff and Price, 1998), cingulate cortex (Scheff and Price, 2001), entorhinal cortex (Scheff et al., 1993), frontal cortex (Scheff et al., 1990), temporal cortex (Scheff and Price, 1993), and precuneus (PreC) (Scheff et al., 2013). Immunohistochemistry studies have also revealed a reduced density of synapses in the frontal cortex (Masliah et al., 1992), entorhinal cortex, and hippocampus (Masliah et al., 1994; Wakabayashi et al., 1994). The latter region also showed reduced post-synaptic drebrin (Counts et al., 2012) and PSD95 as detected by immunoblot in early AD (Sultana et al., 2010). Widespread loss of drebrin, which is involved in synaptic morphology and plasticity (Sekino et al., 2017), has been noted in the superior frontal, superior temporal, visual, inferior parietal, and anterior cingulate cortices whereas pre-synaptic synaptophysin was reduced only in the superior temporal and inferior parietal cortices while synaptotagmin levels were unchanged in all these areas (Counts et al., 2006). Furthermore, a reduction in the density of synapses is also apparent in mild cognitive impairment of the amnestic type in at least the *Cornu Ammonis* 1 (CA1) subregion of the hippocampus (Scheff et al., 2007) and the inferior temporal cortex (ITC) (Scheff et al., 2011).

A subsequent meta-analysis of post-mortem human brain studies confirmed a reduction of synaptic density and loss of synaptic proteins in AD (de Wilde et al., 2016). This analysis also demonstrated that pre-synaptic molecules are more affected than post-synaptic molecules—particularly in the hippocampus compared to cortical areas. Molecules of interest are listed below and a summary of their differential regulation in AD across different brain regions is provided in Table 1. Pre-synaptic markers included those involved in calcium sensing and buffering (e.g., parvalbumin; synaptotagmin—which contrasts with the immunoblot study mentioned above; and synaptophysin); cytoskeletal structures (septin 5 and 7); endocytosis (AP180, dynamin I); small GTPases (rab-3a, -5, and -7); SNAREs (complexins, SNAP25, synaptobrevin, syntaxins, VAMPs); vesicular tethering (synapsin I); and vesicles (SV2, synaptophysin, VGLUTs). Post-synaptic markers included cytoskeletal proteins (drebrin, IRSp53, MAP2, GKAP, synaptopodin); a growth/plasticity marker (GAP43); a metal ion transporter (ZnT-1); protein dephosphorylation (spinophilin); neurotransmitter receptors (GABA, muscarinic, NMDA); and molecular signalling components (CaMKII, PSD95). Common pre-/post-synaptic molecules included those involved in adhesion (catenin β, N-cadherin, NCAM); calcium homeostasis (calbindin, calretinin); cytoskeletal proteins (actin); protein phosphatase (calcineurin); receptors (TrkA); and REDOX signalling (thioredoxin). Overall, there was a loss of pre-, post-, and common-synaptic markers across several brain regions in AD. Interestingly, markers of cytoskeletal organisation were significantly elevated in hippocampal pre-synaptic areas but were decreased in the frontal, cingulate, entorhinal, and temporal cortices. The latter areas also showed increased expression of post-synaptic neurotransmitter receptors. Notably, APP was not examined in these studies despite it’s presence at both pre- and post-synapses (Montagna et al., 2017).

**TABLE 1:**
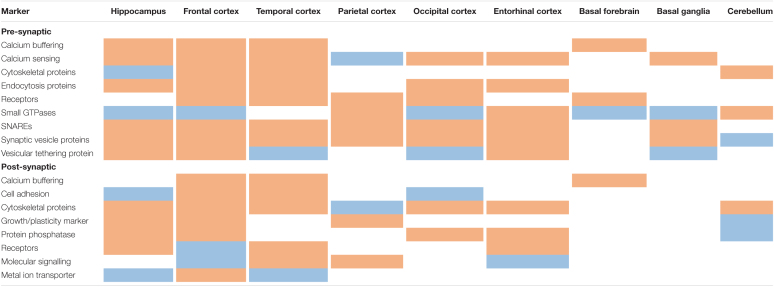
Synaptic markers across differentially affected brain regions in AD.

*Red shading indicates downregulation and blue indicates upregulation in AD; refer to the text for example molecules.*

Early stages of AD are characterised by an apparently compensatory inverse relationship between the increased size of pre-synaptic terminals and the loss of synapses across many cortical and limbic structures (Scheff et al., 1990; Masliah et al., 1994; Scheff and Price, 2003, 2006; Overk and Masliah, 2014). Further, deimpregnation of Golgi-stained neurons followed by Congo red staining has shown that tangle-bearing neurons in the CA1 of the hippocampus exhibit a more extensive dendritic tree than tangle-free neurons (Gertz et al., 1991). It appears that brain areas which normally show an elevated level of synaptic plasticity (for instance the mesial temporal lobe) are associated with increased expression and phosphorylation of tau and are therefore also most prone to the formation of NFTs. Tau dysfunction in these temporal and limbic areas may be further propagated by local neuronal death which would result in increased neuroplasticity demand in adjacent neurons and projection targets which would themselves subsequently be subject to tau dysfunction and the build-up of phosphotau (Mesulam, 1999).

### Expression Microarrays Have Demonstrated the Differential Regulation of Synaptic Genes Across Different Stages of Alzheimer’s Disease

Expression microarray data has demonstrated the downregulation of many synaptic genes in late-stage AD (Berchtold et al., 2013; George et al., 2017; Williams et al., 2021). Berchtold et al. (2013) showed decreased expression of genes involved in synaptic vesicle trafficking and release in the hippocampus. These included SNAREs such as synaptobrevin 2 and SNAP25, and SNARE-related genes, including, synapsin I and II, synaptotagmin 1 and 5, dynamin 1, synaptopodin, rab-3a, syntaxin 6, bassoon, and piccolo, amongst others. Interestingly, several gene transcripts were increased in the hippocampus and entorhinal cortex in AD, including, SNAP23, synaptopodin 2, and synaptobrevin 1. Other pathways that were downregulated in the hippocampus included neuromodulatory peptides (e.g., BDNF, corticotropin releasing hormone, somatostatin, cortistatin, histamine, and tachykinin), voltage-gated ion channels (e.g., Ca^2+^, K^+^, and Na^+^), and an extensive number of transporters, receptors, and enzymes that are required for the synthesis of different neurotransmitters, including glutamate, GABA, acetylcholine, dopamine, glycine, noradrenaline, and serotonin. An earlier investigation using laser capture microdissection of tangle bearing neurons in the CA1 showed upregulation of genes regulating early (rab5) and late endosomes (rab7) which paralleled the downregulation of genes encoding neurotrophin receptors (TrkB; TrkC) over disease progression (Ginsberg et al., 2010).

In contrast, there appears to be an upregulation of genes related to synaptic plasticity before the formation of AD-type neuropathology in the medial frontal cortex of asymptomatic Braak stage II and III brains (Bossers et al., 2010). The same set of genes were only downregulated in the same region following the appearance of Aβ plaques and NFTs in more advanced Braak stages (IV–VI). Among other proteins, Braak stage II was associated with increased expression of several voltage-gated K^+^ channels—regulators of the action potential; GABA receptor subunits; neurotransmitter exocytosis (e.g., SNAREs such as SNAP25, complexin I, synaptotagmins, VAMP7); clathrin heavy chain 1 and protein kinase C—involved in activity-dependent bulk endocytosis; corticotropin releasing hormone; cell adhesion molecule with homology to L1CAM and doublecortin-like kinase 1—proteins involved in neuronal migration and axonal outgrowth; p21-activated kinase 1—involved in activity dependent synaptogenesis; glycine receptor β—involved in the regulation of synaptic connectivity; and stathmin-like 2—a regulator of microtubules during axonal extension. The authors suggested that these expression patterns are indicative of increased synaptic activity and plasticity over the course of Braak stages I–II. Further, they argue that these synaptic alterations are a compensatory response to increased intracellular Aβ and APP fragments containing the Aβ peptide—demonstrated by 4G8 immunoreactivity—which have inhibitory effects on synaptic plasticity in a mouse model and *in vitro* (He et al., 2019). Similarly, synaptoneurosomes derived from early AD cases and individuals with mild cognitive impairment show increased expression of neuroplasticity and synaptic transmission genes such as synaptic vesicle glycoprotein 2A (*SV2A*), growth associated protein 43 (*GAP43*), lipid phosphate phosphatase-related protein type 4 (*LPPR4*), glutamate receptor ionotropic AMPA 2 (*GRIA2*), cholinergic receptor muscarinic 3 (*CHRM3*), and 5-hydroxytryptamine receptor 2A (*HTR2A*) (Williams et al., 2009). Notably, somatostatin was downregulated in the study by Bossers et al. (2010) and also in our recently published RNA sequencing (RNAseq) analysis of the PreC and primary visual cortex (PVC) (Guennewig et al., 2021). The PVC and PreC also showed increased syntaxin binding protein 2 (*STXBP2*)—involved in intracellular vesicle trafficking. The PreC represented a relatively moderately affected area in AD compared to the PVC and showed decreased synaptotagmin 2 (*SYT2*) and *SNAP25*, suggesting that these genes are downregulated relatively soon after their upregulation in the earlier stages of AD.

### Recent Advances in the Understanding of Transcriptomic Signatures of Synaptic Dynamics Highlight Their Dysregulation in Alzheimer’s Disease

Single-cell RNAseq performed in the entorhinal and prefrontal cortices have shown downregulation of excitatory transmission genes (e.g., *SNAP25*; *RIMS1*) in AD (Grubman et al., 2019; Mathys et al., 2019). The latter study found enrichment of regulators of myelination, inflammation, neuronal survival, and global stress response (especially in late-stage disease). However, early stages of AD were associated with the downregulation of both excitatory and inhibitory neuronal genes, which contrasts with findings from the gene microarrays described above. More recently, another study which examined tissues from frontal, temporal, and entorhinal cortices concluded that AD may be subclassed according to either the upregulation or downregulation of genes involved in excitatory synaptic transmission (Neff et al., 2021). This study also corroborated findings from previous RNAseq studies that AD is characterised by dysregulation of genes involved in immune activity, mitochondrial organisation, and myelination in addition to synaptic dysfunction. A single-nucleus RNAseq study also identified impairment of angiogenesis in addition to immune response, myelination, and synaptic signalling in the prefrontal cortex of AD cases (Braak stage ≥ 4) (Lau et al., 2020a). A meta-analysis of transcriptomic data from bulk human AD and mouse tissue showed that the gene signature of synaptic dysfunction in human disease broadly overlapped with the signature seen in mice with a low AD-type pathological burden, which supports the concept of synaptic and neuronal dysfunction as an early event in AD (Wan et al., 2020). Finally, a pre-print of one study which used fluorescence-activated cell sorting of NFT-bearing and NFT-free soma from Braak stage VI AD prefrontal cortex showed NFT-bearing neurons were associated with an upregulation of genes involved in synaptic transmission, including a core set of 63 genes seen across different neuronal subtypes that were enriched for synaptic vesicle cycling (e.g., *SNAP25*; *SYT1* encoding synaptotagmin 1) and transsynaptic signalling (e.g., *NTRK2* encoding the BDNF receptor TrkB) (Otero-Garcia et al., 2020). This result appears consistent with the suggestion that neurons with a high neuroplastic demand are most prone to tangle formation as discussed above.

### Experimental Animal Models of Alzheimer’s Disease Have Demonstrated Reduced Synaptic Plasticity

LTP and LTD—though to a lesser extent—have been intensively investigated (and reviewed) in experimental animal models of AD (Mango et al., 2019). Electrophysiological recordings have shown reduced LTP in certain *APP* mouse strains (Palop et al., 2007; Balducci et al., 2011; D’Amelio et al., 2011; Tozzi et al., 2015). Similarly, *APP*/*PS1* mice show reduced LTP (Trinchese et al., 2008; Calella et al., 2010) and LTD (Chang et al., 2006; Song et al., 2014; Yang et al., 2016). A triple transgenic model (3×Tg-AD mice expressing human *APP*, *PS1*, and *MAPT* mutations) displayed impaired LTP which correlated with intraneuronal Aβ before the formation of Aβ plaques and NFTs (Oddo et al., 2003). Loss of synaptic spines in primary neuronal cultures derived from *APP*/*PS1* mice was shown to cause the loss of coordinated neuronal activity which eventually translated to impaired transmission efficiency and the breakdown of global neuronal network transmission (Kashyap et al., 2019). Conversely, other experimental systems have demonstrated that injury or denervation induced neuroplasticity leads to the upregulation of *APP* (Banati et al., 1993; Wallace et al., 1993; Beeson et al., 1994; Chauvet et al., 1997), the processing of which occurs at synaptic spines (Kamenetz et al., 2003; Priller et al., 2006). It is therefore conceivable that neuroplasticity changes associated with tau pathology act upstream of neuron activity-dependent generation of Aβ and its attendant neuronal network dysfunction—a scenario which is consistent with the synaptic gene microarray data outlined above and which may be especially relevant to sporadic AD (Arnsten et al., 2021). Thus pharmacological rescue of synaptic function represents an area open to pharmacological intervention (Prieto et al., 2017; Jackson et al., 2019).

The ultrastructure and quantitative biochemistry of dendritic spines were recently described in greater detail (Helm et al., 2021). In their investigation the authors combined mass spectrometry, EM, and super-resolution microscopy to annotate 47,000 spines for 110 synaptic proteins in cultured, predominantly glutamatergic, hippocampal neurons. The combination of these data allowed the authors to construct 3D models of the average stubby and mushroom spines. Both types of spines showed similar protein copy numbers and spatial organisation. It would be interesting to determine how the protein, and even lipid, content of spines might change in relation to the timing of Aβ and tau pathology across the AD continuum using similar techniques. For instance, the release of calcium from the endoplasmic reticulum can be up to 10 times greater in AD transgenic mice compared to wild type controls which can impact synaptic structure and function (Goussakov et al., 2010, 2011; Chakroborty et al., 2019).

## Microglia in Alzheimer’s Disease

Microglia constitute a dynamic population of cells in the CNS (Gertig and Hanisch, 2014). They exhibit significant functional plasticity that is also reflected by morphological diversity. It was through the study of morphology that these intrinsic cells were first conclusively implicated in diseases of the CNS (Streit et al., 1988, 2014; Kreutzberg, 1996). Microglia can be distinguished from other cells of the CNS using EM without antibody-based techniques (Savage et al., 2018). Dark microglia are the most recently described type using EM (Bisht et al., 2016). These microglia display signs of oxidative stress, including an electron dense cyto- and nucleoplasm with remodelling of chromatin. They are reactive cells seen in chronic stress, ageing, and in *CXCR1* knock-out and *APP*/*PS1* mice. The morphology of microglia is influenced by many factors, including diet and obesity (Cope et al., 2018); consumption of alcohol (Marshall et al., 2020) and associated hepatic encephalopathy (Dennis et al., 2014); drug consumption (Burkovetskaya et al., 2020); infection and traumatic injury (Giordano et al., 2021); stress (Kreisel et al., 2014); ageing and sex (Brawek et al., 2021); sleep–wake cycles (Nakanishi et al., 2021); sleep deprivation (Wadhwa et al., 2017); and autoimmune and systemic diseases (Aw et al., 2020). Interestingly, microglia may also contribute to sex differences observed in neurodegenerative diseases (Stephen et al., 2019; Chowen and Garcia-Segura, 2021). Most recently in the field of AD, microglia are suggested to contribute to the propagation of Aβ by acting as carriers in brain tissue of young human *APP*-expressing 5×FAD mice (d’Errico et al., 2021).

### Genomic and Transcriptomic Signatures of Microglia in Alzheimer’s Disease and Models of the Disease

The understanding of the molecular signatures of microglia has been expanded considerably over the last decade (Prinz et al., 2019) and includes the development of an atlas of transcriptomic changes in microglia across age, brain regions, and disease pathologies (de Paiva Lopes et al., 2020). RNAseq in mouse models of AD has been used to identify neuroprotective “disease-associated microglia” (DAM) as described by Keren-Shaul et al. (2017) and the “microglial neurodegenerative phenotype” (MGnD) (Krasemann et al., 2017)—both of which are characterised by TREM2 activation and associated with increased APOE expression. Single-cell RNAseq has also uncovered (mouse) disease stage-specific microglial signatures, including two profiles characterised by either type I or II interferon response genes (Mathys et al., 2017). Another RNAseq study in mice showed that immunological imprinting of microglia by either training (a single intraperitoneal injection of lipopolysaccharides) or tolerance (multiple injections) exacerbated or attenuated cerebral Aβ pathology, respectively (Wendeln et al., 2018). It is important that caution is exercised when interpreting results from mouse studies given the key differences between human and mouse single-cell microglial transcriptomes (Chen and Colonna, 2021). It appears that the binding of Aβ to pattern recognition receptors, including the receptor for advanced glycation end-products, nucleotide-binding oligomerisation domain-like, scavenger, formyl peptide, and toll-like receptors—among other mechanisms—is sufficient to cause neurotoxic activation of microglia (Salminen et al., 2009). A number of mechanisms by which neurotoxic microglia exert their effects are proposed including the release of cytokines such as interleukins (ILs), interferons, and tumour necrosis factor (TNF) which are elevated in the cerebrospinal fluid (CSF) of AD patients before the onset of symptoms (Tarkowski et al., 2003) and which have been shown to suppress LTP in rats (Griffin et al., 2006); reduced secretion of trophic factors such as transforming growth factor β (TGFβ) and BDNF (Parkhurst et al., 2013; Heneka et al., 2015); inhibition of mitochondrial respiration through increased nitric oxide (Meda et al., 1995; Poderoso et al., 2019); and phagoptosis of phosphatidylserine presenting neurons (Neniskyte et al., 2011). Furthermore, Baik et al. (2019) reported that exposure to monomeric, oligomeric, or fibrillar Aβ induced microglial activation by shifting metabolism toward aerobic glycolysis via the (mTOR)-hypoxia-inducible factor-1α (HIF-1α) pathway which resulted in the increased production of cytokines such as IL1β and was associated with mitochondrial impairment in microglia. Inhibition of glycolysis in microglia exposed to Aβ by treatment with 2-deoxy-D-glucose (2DG) resulted in reduced IL1β and TNF-α and preserved mitochrondrial function compared to microglia exposed to Aβ only.

Several genome wide association studies (GWAS) have been performed for late-onset AD (Bertram et al., 2008; Harold et al., 2009; Lambert et al., 2009; Hollingworth et al., 2011; Naj et al., 2011), including with concurrent meta-analyses (Lambert et al., 2013; Marioni et al., 2018; Jansen et al., 2019; Kunkle et al., 2019). Many of the common allelic variants which exert only a small effect size on the overall risk of developing AD are those expressed by myeloid cells and microglia in particular (Jones et al., 2015; Pimenova et al., 2018; Bertram and Tanzi, 2019; Andrews et al., 2020; Podleśny-Drabiniok et al., 2020). Other gene categories implicated include those involved in the processing of APP, endosomal-lysosomal vesicle cycling, and lipid and cholesterol metabolism and transport. Gene network and proteomic analyses also suggest that microglia have a role in AD. Gene-regulatory networks have demonstrated the differential regulation of microglial genes governed by DAP12 (encoded by *TYROBP*) and TREM2 signalling pathways (Zhang et al., 2013; Audrain et al., 2021). A study of protein networks in end-stage cortical AD tissue showed enrichment of microglial and astroglial markers suggesting that both are important effectors of cognitive impairment late in the disease course (Seyfried et al., 2017).

*TREM2* rare variants represent the strongest risk factor for developing sporadic late-onset AD after the much more common *APOE* variants. The R47H variant more than doubles the risk of AD (Guerreiro et al., 2013; Jonsson et al., 2013). This polymorphism causes the expression of a truncated protein and a loss of function which results in reduced clearance of Aβ (Yeh et al., 2016). Another related set of genes implicated by GWAS is the *MS4A* family which includes risk polymorphisms in the *MS4A4A* and *MS4A6A* subtypes—the latter being significantly upregulated in both the PreC and PVC in our recent RNAseq report (Guennewig et al., 2021). These proteins are key regulators of cellular activation and levels of soluble TREM2 (Eon Kuek et al., 2016; Deming et al., 2019). The loss of TREM2 facilitates the accumulation of tau pathology but only in the presence of Aβ (Hardy and Salih, 2021; Haass, 2021; Lee et al., 2021). Again, the roles of microglia at different stages deserve attention. For instance, one *in silico* transcriptomic analysis supports the role of TREM2/TYROBP signalling during early stages of disease whereas later stages were associated with soluble TREM2 and nuclear factor κβ (NFκβ) (Ji et al., 2021).

TREM2 and CSF1R are emerging targets for disease-modifying therapeutics in AD and other neurodegenerative diseases (Piccioni et al., 2021). CSF1R is a transmembrane protein expressed by myeloid cells. Its ligands include CSF1 and IL34 (Elmore et al., 2014) and its activation supports the development of myeloid cells in mice (Lei et al., 2020). The exposure of 5×FAD mice (expressing three *APP* and two *PSEN1* human mutations) to CSF1R inhibitors show reduced proliferation of microglia which are characterised by a shift to an anti-inflammatory profile and are associated with reduced neuronal loss (Spangenberg et al., 2016). Later work also showed CSF1R inhibitors prevented the formation of parenchymal Aβ plaques, but not CAA (Spangenberg et al., 2019). TREM2 is a transmembrane immunoglobulin expressed by microglia (Ulland and Colonna, 2018) and as mentioned is implicated in the pathogenesis of AD (Gratuze et al., 2018). Notably, there is controversy surrounding the expression of *TREM2* by human microglia as the recruitment of peripheral myeloid cells may have been underappreciated to date (Fahrenhold et al., 2018). Notwithstanding this caveat, it appears that an effective microglial response to Aβ is TREM2-dependent and limits the extent of phosphotau pathology (Lee et al., 2021). The loss of function of TREM2 (as seen with the R47H mutation) results in reduced binding affinity to APOE compared to wild-type protein, resulting in reduced clustering of microglia at Aβ plaques and increased Aβ load (Krasemann et al., 2017). Interestingly, *TREM2*^(+/–)^ deficiency is associated with increased Aβ plaques and phosphotau-positive dystrophic neurites compared to complete knock-out of *TREM2*^(–/–)^ in AD mice, demonstrating a complex relationship between microgliosis and plaque-associated neurofibrillary pathology (Delizannis et al., 2021). More broadly, the loss of TREM2 function is also associated with reduced microglial survival (McQuade et al., 2020) and impaired lipid metabolism and thus represents a druggable target of particular interest (Deczkowska et al., 2020; Lewcock et al., 2020; Hardy and Salih, 2021; Haass, 2021).

Similarly, a loss of APOE function is associated with an impaired microglial response to AD pathology (Pimenova et al., 2017). It appears the interaction between APOE and TREM2 is required for an adequate microglial response to Aβ pathology (Shi and Holtzman, 2018; Nguyen et al., 2020). Single-cell RNAseq of human microglia demonstrated the loss of a subset of microglia which highly express *APOE* and *TREM2* in the context of AD (Olah et al., 2020). Whilst APOE deficiency in *APP/PS1* mice was associated with reduced density of Aβ plaques, remaining plaques showed reduced compaction, a loss of microglial clustering around plaques, worsened NFD, and a significant downregulation of immune-related genes (Ulrich et al., 2018) and others such as *Itgax* and *Cst7*—genes which are highly expressed in DAM (Keren-Shaul et al., 2017). Unexpectedly, the complete knock-out of microglia-specific *APOE* in 5×FAD mice did not alter plaque load, number of microglia, or clustering of microglia but was associated with increased average plaque size (Henningfield et al., 2022)—notably in this study, astroglial APOE is still present. The *APOE ε4* allele contributes to the disruption of glial homeostatic functions (Fernandez et al., 2019). *APOE4* carriers show an increased number of activated microglia compared to *APOE3* controls (Egensperger et al., 1998) and the expression of human *APOE4* increases reactive microglia with dystrophic processes around Aβ plaques which are larger in size compared to human *APOE3*-expressing mice (Rodriguez et al., 2014). It is likely that other microglial pathways, such as the activation of the inflammasome with increased downstream caspase-1 and IL1β activity (Ising et al., 2019) or the hypersecretion of extracellular vesicles (Clayton et al., 2021), also contribute to the accumulation of tau pathology. The cross talk and role of other glial cells over the course of AD will also have to be closely investigated for a more complete understanding of the disease and may open new avenues of therapies. For instance, astrocytic IL3 has been shown to promote the neuroprotective activation of microglia (McAlpine et al., 2021).

Distinct microglial transcriptional profiles are associated with Aβ or tau pathology (Gerrits et al., 2021; Lemprière, 2021). Gerrits et al. (2021) described 13 transcriptional subclusters of microglia in post-mortem AD tissues from the PVC and occipitotemporal cortex (fusiform gyrus). There were two subclusters labelled “AD1” and “AD2” which were of particular interest. AD1 microglia were most prominent in cases with only Aβ pathology in which the microglia correlated with Aβ load and localised to Aβ plaques. This correlation was absent in AD cases exhibiting both Aβ and tau pathology. AD2 microglia occurred in AD cases with both Aβ and tau pathology in which these microglia correlated with phosphotau. AD1 microglia showed enrichment of markers of phagocytosis and activation, including *ITGAX*, *LPL*, *GPNMB*, *MYO1E*, and *SPP1*. AD2 microglia showed enrichment of homeostatic genes (e.g., *CX3CR1* and *P2RY12*) and a number of neuron-related genes such as *GRID2*, *ADGRB3*, and *DPP10*. Among the eleven other subclusters was one enriched for genes of proliferation (e.g., *TOP2A* and *MKI67*), another for markers of cellular stress (e.g., early response genes—*FOS* and *JUNB*; heat-shock genes—*HSPA1A* and *HSPA1B*), and homeostatic subclusters which were inversely correlated with the load of Aβ or tau pathology. The correlation of microglial genes with Aβ pathology was noted recently by us (Tsatsanis et al., 2021) and others beforehand (Matarin et al., 2015). The latter genome-wide expression analysis also demonstrated correlations between genes of synaptic plasticity to tau pathology. The close association between Aβ and activated microglia prior to tau deposition has now been noted in post-mortem neuropathological studies (Sheffield et al., 2000; Eikelenboom et al., 2010; Paasila et al., 2020), tau transgenic mice (P301S) (Yoshiyama et al., 2007), *in vitro* (Hopp et al., 2018), and in PET imaging studies (Zou et al., 2020; Pascoal et al., 2021).

### Post-mortem Immunohistopathology Highlights the Degeneration of Microglia in Advanced Alzheimer’s Disease

A characterisation of the morphological subtypes of microglia in AD and their relationship to Aβ and NFD across differentially affected regions of the AD brain was performed in our lab (Paasila et al., 2019). In this study, a major feature of cortical AD tissue was the dramatic reduction in the number of healthy ramified Iba1-positive microglia in the ITC—a severely affected area of the AD brain in terms of neuronal loss and cortical atrophy. The observed reduction in Iba1 and its fragmented distribution can be assumed to have serious implications for the motility (Franco-Bocanegra et al., 2019), membrane ruffling, and phagocytic capacity of the microglial cell population given its role as an actin crosslinking protein essential for actin bundling (Bartles, 2000; Ohsawa et al., 2000, 2004; Sasaki et al., 2001) and its enrichment *in vitro* during phagocytosis of full-length tau oligomers (Das et al., 2020). Interestingly, the ITC showed a significantly increased density of activated microglia in preclinical AD cases only (cognitive controls with Alzheimer-type pathological changes at post-mortem—CAc). Activated microglia with reduced branching also increased with age as seen in an earlier (Davies et al., 2017) and recent post-mortem investigation (Casaletto et al., 2021). Further, the density of clusters of activated microglia was significantly higher in mildly affected areas of the AD brain such as the primary motor cortex (PMC) and PVC. Findings in the PMC also demonstrated the clustering of activated microglia around Aβ plaques ahead of the formation of dystrophic neurites (Paasila et al., 2020). However, the total percentage of microglia associated with a cluster was <2% and only a minority of Aβ plaques were associated with a cluster—the proportion of which decreased in a stepwise fashion from mildly to severely affected regions. Lastly, the internalisation of synaptophysin-positive pre-synapses was found to be significantly elevated in the superior frontal cortex of AD cases compared to both CAc and controls without AD-type pathology using super-resolution microscopy (Paasila et al., 2021). Synaptophysin is one of the most severely affected synaptic markers in AD (Reddy et al., 2005). It would be of interest to determine if the microglia observed in our latter study were targeting viable neurons or those already marked for removal or undergoing apoptosis.

The concept of protective microglial activation early in the disease time course followed by degeneration associated with a loss of homeostatic function during the end-stage of disease is increasingly appreciated (Navarro et al., 2018; Bennett and Liddelow, 2019; Schwabe et al., 2020; Streit et al., 2020; Chatila and Bradshaw, 2021). A previous investigation has highlighted the stark contrast between *APP*-based animal models which show strong activation of microglia and post-mortem human hippocampus which contrastingly showed a weak microglial response (Sanchez-Mejias et al., 2016)—similarly described in cortical tissue by us (Paasila et al., 2019). Sanchez-Mejias et al. (2016) also presented results from an *in vitro* investigation showing that the soluble fraction of phosphotau is responsible for driving the degeneration observed in microglia. The loss of ramified cells in AD observed by us has been validated in a larger cohort by others—albeit without the expected loss of branching complexity of residual cells (Franco-Bocanegra et al., 2021)—and has similarly been associated with AD-type neuropathology in the brains of people with Down’s syndrome (Martini et al., 2020). Dystrophic microglia were also associated with NFD in our investigations (Paasila et al., 2020) and in others’ (Streit et al., 2009). However, in our study brain pH showed a stronger correlation with dystrophic microglia than disease status (Paasila et al., 2019), highlighting the importance of agonal factors as a cause of phenotypic changes in microglia. More broadly, this would have important implications for –omics studies using post-mortem human tissue if the prominence of the immune signalling pathways is a residual of brain pH (Monoranu et al., 2009; Durrenberger et al., 2010).

## Lipids in Alzheimer’s Disease

### Genome Wide Association Studies Have Identified Lipid Metabolism as a Major Risk Category in Alzheimer’s Disease

The plasma membrane and the internal cellular endomembranes are mainly composed of lipids. There are many factors which affect lipid metabolism, including, age, sex, genetics, diet, and physical activity (Chew et al., 2020). Lipids are transported throughout the body as lipoproteins, molecules which have a hydrophobic centre of cholesterol, esters, and triglycerides surrounded by amphipathic phospholipids with the addition of apolipoproteins. Lipids influence the trafficking and proteolytic cleavage of key proteins in AD and their propensity to self-aggregate (Di Paolo and Kim, 2011). As discussed above, AD GWAS results can be divided into anomalies in neuroimmune function, endocytosis, and lipid (and cholesterol) metabolism (Pimenova et al., 2018; Vogrinc et al., 2021). The genetics underlying lipid metabolism are of particular interest as the dissociation of phospholipids may be the defining feature of neurodegeneration in AD (Hardy, 2017). The major drivers of the enrichment of lipid metabolic pathways in GWAS (excluding *APOE*) include *ABCA7*, a key regulator of cellular cholesterol, and apolipoprotein genes *APOM* and *APOA5* (Kunkle et al., 2019). Additional genes identified from GWAS that are involved in lipid metabolism include other apolipoproteins (*APOA1*, *APOA4*, *APOC1*, *APOC2*, *APOC3*, *APOC4*, and *APOJ*); an intracellular cholesterol transporter (*NPC1*) and membrane-bound cholesterol pumps (*ABCA1*, *ABCA2*, and *ABCG4*); phospholipid transporters (*ATP8A1*, *ATP8A2*, *ATP8B4*, *PCTP*, and *PLCG2*); intracellular lipid receptors (*OSBPL7* and *OSBPL9*); a high-density lipoprotein receptor (*SCARB1*); hepatic lipase (*HTGL*) and endothelial lipase (*LIPG*); MAL-like protein (*MALL*)—a component of cholesterol rafts; sterol O-acyltransferase-1 (*SOAT1*)—involved in the synthesis of fatty acids and cholesterol esters; and a fatty acid transporter (*SLC27A4*) (Jones et al., 2010).

### Alzheimer’s Disease Is Characterised by Broad Dysregulation of Lipid Compounds in Blood Plasma and Across Different Regions of the Brain

Alzheimer’s disease is characterised by significant dysregulation of fatty acid (Snowden et al., 2017) and lipid metabolism (Wood, 2012; Touboul and Gaudin, 2014; Wilkins and Trushina, 2017; Kao et al., 2020). For example, a study by Sáiz-Vazquez et al. (2020) showed AD is associated with high serum LDL-cholesterol. Metabolomic analyses of two brain autopsy series (Baltimore Longitudinal Study of Aging and the Religious Orders Study) showed that whilst cholesterol is unchanged in AD brains, cholesterol precursors are reduced and decomposition metabolites (including non-enzymatically generated cytotoxic oxidised forms—oxysterols) are increased (Varma et al., 2021). However, at least one other study showed elevated cholesterol levels in cortical samples of AD brains (Lazar et al., 2013). Glycerolipids such as monoacylglycerol and diacylglycerol are increased in the frontal cortex and blood plasma early in the pathogenesis of AD (Chan et al., 2012; Wood et al., 2015). Conversely, triglycerides—the predominant glycerolipid in the body—are reduced in plasma of mild cognitive impairment and AD (Bernath et al., 2020). Other plasma lipidomic studies have also demonstrated reduced levels of most cholesteryl esters (Liu et al., 2021)—particularly long chain species (Proitsi et al., 2015)—in mild cognitive impairment and AD. Glycerophospholipids are the major constituent of the cell membrane (van Meer et al., 2008). Glycerophospholipids such as phosphatidylcholine, phosphatidylethanolamine, ethanolamine plasmalogen, and cardiolipin are significantly decreased in the AD cortex, with certain species inversely correlated to AD severity (Nitsch et al., 1992; Ginsberg et al., 1995; Pettegrew et al., 2001). Blood serum also shows significant reduction of glycerophospholipid content (Mapstone et al., 2014; Whiley et al., 2014). Lastly, sphingolipids including sphingomyelins (Varma et al., 2018) and ceramides (Filippov et al., 2012) are increased in AD brains, but reduced in CSF (Fonteh et al., 2015). Other sphingolipids including sphingosine 1-phosphate (He et al., 2010), sulphatides (early in the disease) (Cheng et al., 2013), and gangliosides (Ariga, 2017) are reduced in AD brains. Notably, plaque-associated lipids have been successfully visualised (Kiskis et al., 2015). Lipid deposits co-localised with Thioflavin-S-positive Aβ plaques and showed either lamellar structure or occurred as coalescing macro-aggregates up to ∼25 μm in size. Conversely, diffuse plaques did not show the same association with lipid structures. Aβ oligomers have been shown to bind to the cell membrane to cause rupture or pore formation capable of eliciting apoptosis associated with mitochondrial death (Deshpande et al., 2006).

### Experimental Models of Alzheimer’s Disease Suggest That Abnormal Lipid Metabolism, Neuropathological Tissue Changes and Cognitive Impairment Are Linked

There are several experimental systems which have been used to elucidate the mechanisms by which lipid and related pathway alterations cause neurodegeneration. *APP*/*PS1* mice have demonstrated a connection between lipid derangements and cognitive impairment (Zhang et al., 2020). Serum triglycerides readily cross the blood-brain-barrier and contribute to the impairment of memory and learning (Banks et al., 2018) possibly by the disruption of the NMDA component of hippocampal LTP (Farr et al., 2008). Cholesteryl esters—the storage product of excess cholesterol—have been identified as an upstream regulator of tau pathology using induced-pluripotent stem cell (iPSC) lines carrying *APP* mutations (van der Kant et al., 2019). In this study the reduction of cholesteryl esters reduced phosphotau by increasing proteasome activity. Other studies have examined the role of lipid receptors. For instance, the deletion of low-density lipoprotein-related protein 1 (LRP1) in adult mouse forebrain neurons lead to global defects in brain lipid metabolism, including decreased cholesterol, sulphatides, ceramides, and glycerolipids which culminate in synaptic loss (Liu et al., 2010). In line with this, a recent report demonstrated that the overexpression of low-density lipoprotein receptor (LDLR) markedly reduced APOE and attenuated NFD and neurodegeneration in P301S mice (Mendiola et al., 2021; Shi et al., 2021)—similarly demonstrated in an earlier study using *APP*/*PS1* mice (Kim et al., 2009b). In addition, changes to lipid content in PLB4 mice expressing human β-secretase 1 (hBACE1) have shown brain region-specific vulnerabilities to lipid dysregulation such as in the hippocampus and hypothalamus, suggesting that changes to lipid content may leave certain brain regions more susceptible than others (Dey et al., 2020).

### The Interrelationships Between Synapses, Microglia, and Lipids in Alzheimer’s Disease

Brain cholesterol dysmetabolism is related to synaptic dysfunction in a number of neurological diseases (Petrov et al., 2016), including AD (Petrov et al., 2017). Microglia have an important role in lipid metabolism (Loving and Bruce, 2020). Microglia are capable of sensing excess saturated fats and modulating hypothalamic control of energy metabolism (Folick et al., 2021). They are also sensitive to fatty acids derived from the body’s microbiota through which they may modulate Aβ deposition (Colombo et al., 2021). Valdearcos et al. (2014) reported that microglia in the murine mediobasal hypothalamus undergo inflammatory activation in response to excess dietary saturated fatty acids. More closely related to AD, the Aβ-induced reduction in sphingosine kinase, an important regulator of lipid secretion from neurons, is associated with defective microglial phagocytosis and is also associated with increased expression of the cytokines (e.g., TNFα, IL1β, IL6, and inducible nitric oxide synthase) and reduced IL4, TGFβ, and arginase-1 (Lee et al., 2018). They are also essential for remyelination following injury via TREM2-dependent lipid droplet biogenesis—a process which incidentally involves the esterification of cholesterol (Gouna et al., 2021) and which may be relevant given the potential of age-related myelin degeneration as a driver of Aβ pathology (Depp et al., 2021). It also appears that phagocytic activity governed by TREM2 transcriptionally regulates cholesterol transport and metabolism, whereby TREM2-deficient microglia capably phagocytose myelin debris but fail to clear excess cholesterol resulting in the accumulation of cholesteryl esters (Nugent et al., 2020).

**FIGURE 1:**
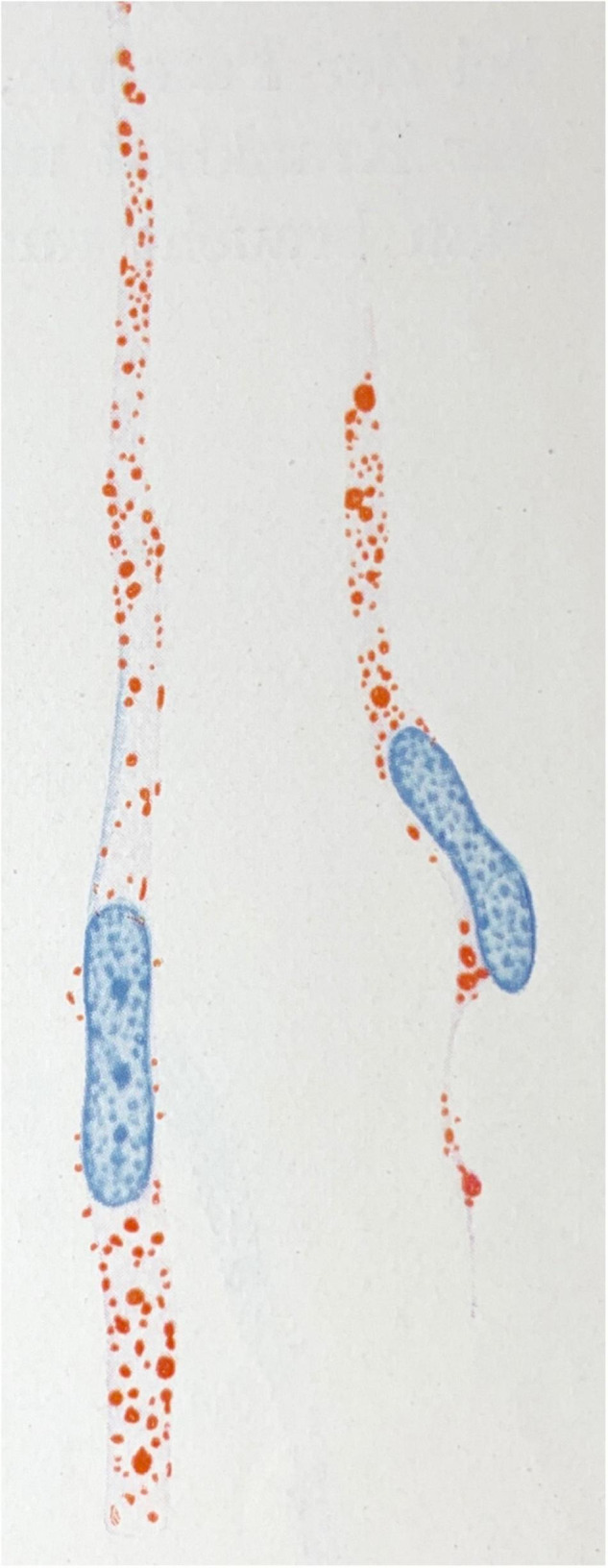
Microglial rod cells rich in lipoid material (scarlet red staining; Sudan IV). Drawing taken from Spielmeyer (1922).

**FIGURE 2:**
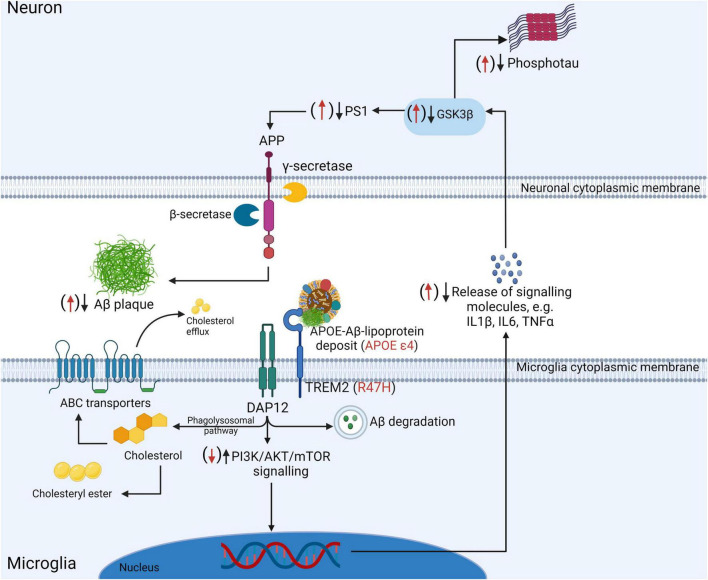
Under physiological conditions TREM2 facilitates the phagocytosis of APOE-lipoprotein-bound Aβ. Lipid molecules are processed by the intracellular phagolysosomal system to yield free cholesterol which may be stored internally as cholesteryl esters or secreted by ATP-binding cassette (ABC) transporters. Aβ may be degraded in autophagic vacuoles, via the ubiquitin-proteasomal system, or by any of a number of Aβ-degrading proteases including the metalloendopeptidase and matrix metalloproteinase families of catabolic enzymes. Furthermore, DAP12 activation following TREM2 binding results in the activation of the PI3K/AKT/mTOR signalling cascade which reduces the secretion of signalling molecules such as IL1β, IL6, and TNFα which in turn is associated with reduced activation of intraneuronal GSK3β—an important protein kinase responsible for tau phosphorylation and which is also involved in the activation of γ-secretase through its interaction with presenilin 1. The R47H loss of function mutation in TREM2 and the APOE ε4 allele (both indicated in red) represent two factors which impair Aβ and lipid processing by microglia. Abnormal TREM2/DAP12 signalling may result in reduced PI3K/AKT/mTOR signalling and disinhibition of IL1β, IL6, and TNFα secretions, leading to increased intraneuronal phosphotau and the exacerbation of Aβ accumulation (indicated by the red arrows). Created with BioRender.com.

Indeed, “lipid-droplet-accumulating microglia” (LDAM) accrue with age in humans and mice and exhibit defective phagocytic capability, overproduce reactive oxygen species, and secrete excess signalling cytokines such as TNFα, IL1β, and IL6 (Marschallinger et al., 2020; cf., Figure 1). The accumulation of lipid droplets have also been noted in *APP*/*PS1* chimeric mice xenografted with wild-type or R47H-TREM2 mutant iPSCs (Claes et al., 2021). In the latter study, the R47H mutation resulted in reduced clustering of microglia at plaques but critically did not impair the formation of lipid droplets within individual cells. Microglia also appear active in the regulation of cholesterol-modulated phosphotau pathology through activation and phagocytic pathways (Nanjundaiah et al., 2021). Excess cholesterol and other lipids are detected by microglial TREM2 (amongst other receptors) when complexed with apolipoproteins (such as APOE or APOJ) which in turn activates the PI3K/AKT/mTOR signalling cascade (Peng et al., 2010) which is linked to the inhibition of GSK3β (Hermida et al., 2017)—an important mediator of intraneuronal tau hyperphosphorylation (Hooper et al., 2008). The remote attenuation of neuronal kinases potentially occurs through reduced expression of the aforementioned TNFα, IL1β, and IL6 as described in TREM2-overexpressing P301S transgenic mice (Jiang et al., 2016)—refer to Figure 2 for an outline of this process. Further, autophagy-lysosomal dysfunction appears sufficient to cause lipid droplet formation in microglia, increased IL1β and TNFs, and is associated with enhanced intraneuronal tau pathology and substantial synaptic degeneration (Xu et al., 2021).

Several studies have identified distinct transcriptomic profiles in murine DAM for pattern recognition, lipid metabolism, and lysosomal pathways. Keren-Shaul et al. (2017) demonstrated an increased presence of DAM characterised by intracellular Aβ particles and elevated Lpl, Cst7, and Cd9—molecules involved in lipid uptake and phagocytosis—in a 5×FAD model of AD. Ofengeim et al. (2017) reported increased surface-bound enzyme cholesterol 25 hydroxylase (Ch25h) and Cst7 levels in microglia from *APP*/*PS1* mice which were associated with reduced phagocytic capacity and lysosomal pathway impairment. Recently, Lau et al. (2020b) reported that IL33 injection in an *APP*/*PS1* mouse model resulted in Aβ-plaque-associated microglia acquiring the DAM transcriptomic profile with increased levels of Apoe, Axl, Cst7, Lpl, and Trem2 and which was associated with increased clearance of Aβ. Together these findings demonstrate a close relationship between microglial lipid metabolism and phagocytosis.

The role of the complement pathway in microglia-mediated clearance of synapses in certain CNS regions is well established (Stevens et al., 2007). Graeber et al. (1988) first reported a striking upregulation of CR3 complement receptor expression in activated microglia under conditions of synaptic plasticity, suggesting the non-immunological involvement of the complement pathway in neuronal repair following axotomy. More recently, Lim and Ruthazer (2021) observed microglial trogocytosis of axons that was enhanced by neuronal expression of complement receptors *in vivo* in the *Xenopus laevis* retinotectal circuit. However, Weinhard et al. (2018) did not observe changes to trogocytosis following knockout of CR3 in mice, which suggested to the authors no role for complement pathway in trogocytosis. Mutations in the sushi repeat protein X-linked 2 (SRPX2) protein expressed by neurons have been identified in controlling complement pathway-mediated synapse elimination by microglia. Cong et al. (2020) reported that SRPX2 was able to block C1q activation and thereby inhibit the classical complement-mediated elimination of synapses. They also reported that SRPX2^(–/Y)^ knockout mice have increased C3 deposition and microglial synapse engulfment in the dorsal lateral geniculate nucleus (dLGN). Avila-Martin et al. (2017) further reported that albumin-hydroxyoleic acid complex (A-HOA) promoted sensorimotor function recovery in rats with spinal cord injury (SCI) by upregulating several genes including SRPX2, suggesting that SRPX2 may play a role in reestablishing vascularisation and recovering synapse loss associated with SCI. In another study, Cong et al. (2021) showed that the deletion of C1q resulted in a persistent decrease in microglia-mediated synapse elimination and engulfment in the visual cortex. In our RNAseq study, *C4A* (encoding C4) and *C5AR1* expression was increased in the PreC, with the latter also increased in the PVC (Guennewig et al., 2021). Interestingly *CFHR5* (involved in the regulation of the alternative complement pathway) expression was substantially reduced in the PVC.

**FIGURE 3:**
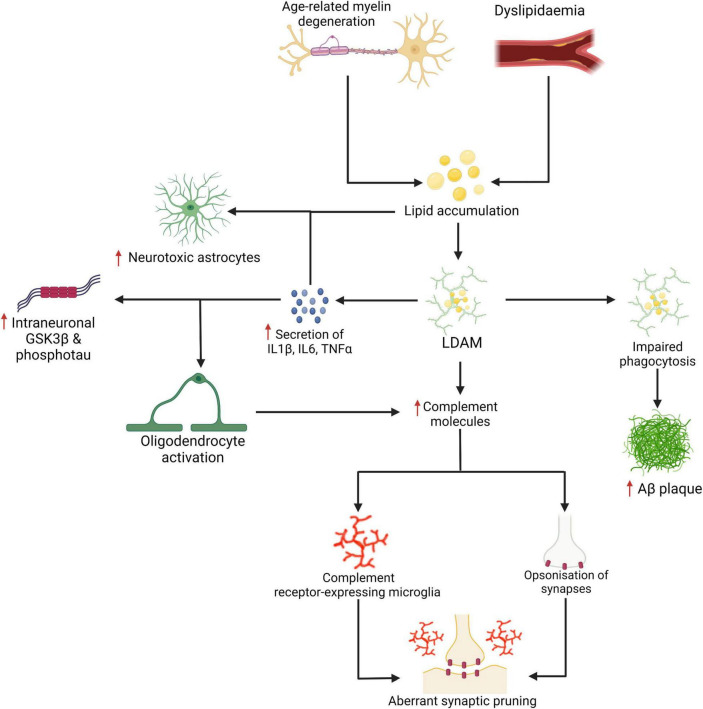
Age-related myelin degeneration and dyslipidemia represent two risk factors for the accumulation of saturated fats and other lipids in the brain. These lipids may be internalised by microglia and stored as cholesteryl esters. Lipid-droplet-accumulating microglia (LDAM) accumulate with age and are associated with increased secretion of IL1β, IL6, TNFα, complement molecules, and show impaired maturation of phagosomes and reduced phagocytic capacity. As demonstrated in Figure 2, the increased secretion of certain cytokines and impaired phagocytosis are linked with more severe neuropathological changes. The signalling cytokines are also implicated in the activation of oligodendrocytes (which may be a source of excess complement deposition) and the neurotoxic activation of astrocytes (for which saturated fats are a sufficient driver). Increased expression and deposition of complement proteins may represent a key event in the targeting of synapses by microglia in AD. Created with BioRender.com.

A number of recent studies highlight the impact of lipid metabolism on complement signalling pathways. For instance, complement factor C3 and C4 levels have been associated with cardiometabolic risk factors such as obesity and insulin resistance (Arias de la Rosa et al., 2020), fat distribution (Fu et al., 2020), and metabolic syndrome and diabetes (Copenhaver et al., 2020) in humans. In animal studies, findings have also demonstrated that peptide antagonists of complement receptors C3aR and C5aR inhibited diet-induced obesity, adipose inflammation, and metabolic dysfunction in rats and ameliorated inflammatory responses in murine macrophages (Lim et al., 2013)—a topic reviewed by Barbu et al. (2015). In the brain, Madore et al. (2020) reported that low omega-3 fatty acid intake altered the expression of complement cascade proteins both in microglia and at the synapse and exacerbated spine phagocytosis. In AD, clearance of Aβ has been shown to be significantly impacted by the presence of lipids as well as by lipid metabolism which may indirectly inhibit complement-mediated microglial clearance of Aβ plaques. Time-lapse atomic force microscopy has demonstrated that the presence of cholesterol in the cell lipid bilayer significantly enhances Aβ_42_ aggregation (Banerjee et al., 2021). Lipid membranes containing cholesterol promote Aβ_42_ aggregation via a heterogeneous nucleation pathway (Habchi et al., 2018). Finally, TREM2 may exert toxic effects later in the disease through a failure of the PI3K/AKT/mTOR pathway and increased IL1β, IL6, TNFα, and complement proteins despite showing protective effects earlier in the disease through the clearance of Aβ. Linnartz-Gerlach et al. (2019) demonstrated that aged TREM2 knock-out mice had lower transcription of C1qa, C1qb, C1qc, C3, and C4b. Thus the reactivation of complement-mediated synaptic pruning is a distinct possibility in AD (Stephan et al., 2012; Heppner et al., 2015; Brucato and Benjamin, 2020; Gomez-Arboledas et al., 2021), a scenario that has been also observed in human mutant *APP* mice (J20) (Hong et al., 2016). Refer to Figure 3 which presents a basic mechanism linking LDAM and aberrant synaptic elimination by microglia which may be mediated by increased complement deposition. On a positive note, it has been shown that age-related microglial activation can be reduced by increased physical activity given a recent retrospective study which found associations between late life physical activity, reduced microglial activation, reduced synaptic loss, and better cognitive outcomes (Casaletto et al., 2021). Although microglia have been a focus here, the role of other glial cells bear careful consideration. For instance, oligodendrocytes may be an important source of complement deposition in AD (Hosokawa et al., 2003) and astrocytes may exert neurotoxic effects following exposure to saturated lipids (Guttenplan et al., 2021) or microglial IL1α, TNF, and C1q (Liddelow et al., 2017). Indeed, single-nucleus RNAseq has demonstrated diverse astrocytic signatures in AD characterised by the enrichment of inflammatory, proteostatic genes, and metal ion homeostatic genes (Smith et al., 2021). Notably, the involvement of metal ions in AD have recently been reviewed by Lei et al. (2021).

## Conclusion

**FIGURE 4:**
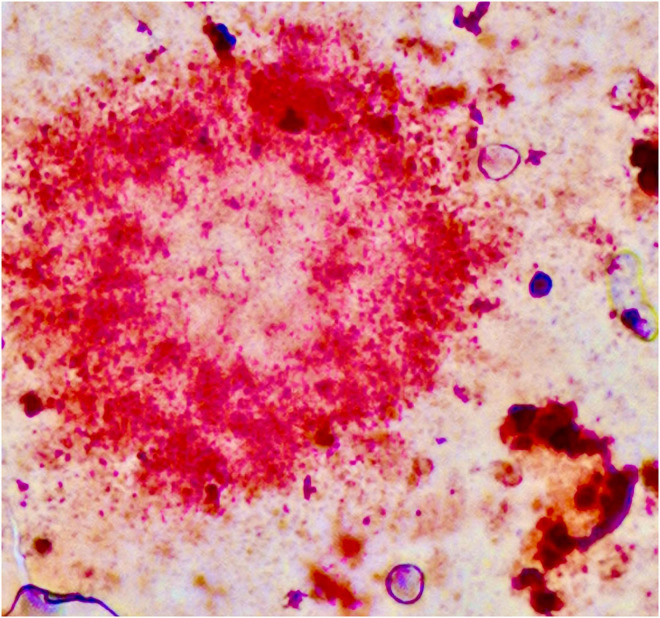
Amyloid plaque stained using the Herxheimer technique (Sudan IV). Significant amounts of lipids are found inside the plaque and in neighbouring glial cells. One cell probably representing a microglial cell is shown in the lower right. Formalin-fixed brain tissue, frozen section. Photograph taken by the authors (MBG): 20× oil primary magnification. Tissue section from Alois Alzheimer’s laboratory (Alzheimer, 1911; Graeber et al., 1997).

A purely biochemical, cell-autonomous view of AD—in which neurotoxic species of Aβ and phosphotau are solely responsible for neurodegeneration—has been described as “*untenable*” (De Strooper and Karran, 2016). Our own work suggests that the activation of microglia early in the disease time course is neuroprotective. The basic science on microglial biology is accelerating but remains a knowledge gap in our understanding of the pathogenesis of AD. For instance, it appears that at least some of the lipid associations seen in GWAS manifest through the dysfunction of microglia rather than simply the disruption of cellular membranes or neuronal function. The activities of microglia in AD represents one avenue for therapeutic intervention (Takata et al., 2021). However, given the complexity of the disease it is not unreasonable to expect that personalised treatment (Gauthier et al., 2018) or a combination of therapies targeting several lines of pathological processes—analogous to the therapeutic strategies used in cancer, HIV, tuberculosis, and cardiovascular disease—will be necessary for its successful management (Cummings et al., 2019; Salloway et al., 2020; Ju and Tam, 2021). To add complexity, further consideration for mixed pathology will also be essential in the management of patients in whom the dementing syndrome is caused by the cumulative effect of different disease states (Kapasi et al., 2017; Thomas et al., 2020) as AD neuropathology infrequently occurs in isolation (Schneider et al., 2007; James et al., 2012; Rahimi and Kovacs, 2014; Brenowitz et al., 2017; Boyle et al., 2018; De Reuck et al., 2018; McAleese et al., 2021). Lastly, the accumulation of lipids in amyloid plaques as well as in glial cells including microglia (Figure 4) deserves further study—age-related myelin degeneration and synaptic terminal membranes could be two relevant sources.

## Author Contributions

All authors listed have made a substantial, direct, and intellectual contribution to the work, and approved it for publication.

## Conflict of Interest

The authors declare that the research was conducted in the absence of any commercial or financial relationships that could be construed as a potential conflict of interest.

## Publisher’s Note

All claims expressed in this article are solely those of the authors and do not necessarily represent those of their affiliated organizations, or those of the publisher, the editors and the reviewers. Any product that may be evaluated in this article, or claim that may be made by its manufacturer, is not guaranteed or endorsed by the publisher.
